# Understanding variations in health insurance coverage in Ghana, Kenya, Nigeria, and Tanzania: Evidence from demographic and health surveys

**DOI:** 10.1371/journal.pone.0201833

**Published:** 2018-08-06

**Authors:** Hubert Amu, Kwamena Sekyi Dickson, Akwasi Kumi-Kyereme, Eugene Kofuor Maafo Darteh

**Affiliations:** 1 Department of Population and Behavioural Sciences, School of Public Health, University of Health and Allied Sciences, Hohoe, Ghana; 2 Department of Population and Health, University of Cape Coast, Cape Coast, Ghana; RTI International, UNITED STATES

## Abstract

**Background:**

Realisation of universal health coverage is not possible without health financing systems that ensure financial risk protection. To ensure this, some African countries have instituted health insurance schemes as venues for ensuring universal access to health care for their populace. In this paper, we examined variations in health insurance coverage in Ghana, Kenya, Nigeria, and Tanzania.

**Methods:**

We used data from demographic and health surveys of Ghana (2014), Kenya (2014), Nigeria (2013), and Tanzania (2015). Women aged 15–49 and men aged 15–59 years were included in the study. Our study population comprised 9,378 women and 4,371 men from Ghana, 14,656 women and 12,712 men from Kenya, 38,598 women and 17,185 men from Nigeria, and 10,123 women and 2,514 men from Tanzania. Bivariate and multivariate techniques were used to analyse the data.

**Results:**

Coverage was highest in Ghana (Females = 62.4%, Males = 49.1%) and lowest in Nigeria (Females = 1.1%, Males = 3.1%). Age, level of education, residence, wealth status, and occupation were the socio-economic factors influencing variations in health insurance coverage.

**Conclusions:**

There are variations in health insurance coverage in Ghana, Kenya, Nigeria, and Tanzania, with Ghana recording the highest coverage. Kenya, Tanzania, and Nigeria may not be able to achieve universal health coverage and meet the sustainable development goals on health by the year 2030 if the current fragmented public health insurance systems persist in those countries. Therefore, the various schemes of these countries should be harmonised to help maximise the size of their risk pools and increase the confidence of potential subscribers in the systems, which may encourage them to enrol.

## Introduction

Universal health coverage (UHC) is the expectation that all persons shall obtain the preventive, promotive, rehabilitative, curative, and palliative health services they require without experiencing financial challenges in paying for such services [1]. The attainment of UHC promotes enjoyment of the highest attainable standard of health, which is considered one of the basic human rights of every individual regardless of their religion, race, social or economic conditions, and political beliefs [2,3]. The realisation of the utmost attainable standard of health is, however, not possible without health systems that function appropriately and health financing mechanisms that ensure financial risk protection, especially for the poor [4–6]. Countries across the globe have, therefore, adopted different health financing mechanisms, including social health insurance, to ensure universal access to quality and basic health care for their populace [7].

Whereas developed countries such as Australia and Canada have been successful in adequately financing the health needs of their populace through a combination of public and private health insurance systems [8], health care accessibility through health insurance in developing countries remains limited due to socio-economic challenges [9]. These challenges are especially experienced in Africa, a continent known to have a strong tendency for risk distribution across populations and time [10]. Thus, several African countries, including Ghana, Kenya, Nigeria, and Tanzania, are currently implementing various health insurance options at the general population level, most of which are public schemes [11–15].

To ensure that every Nigerian resident has easy access to health services, the National Health Insurance Scheme (NHIS) of Nigeria, upon its promulgation in 1999 (Act 35), introduced various programmes that cover varied sectors of the country [16]. These comprised an urban self-employed social health insurance programme, formal sector social health insurance programme, rural community social health insurance programme, children under five social health insurance programme, prison inmates social health insurance programme, disabled persons social health insurance programme, the police, armed forces, and other uniformed services health insurance programme, the vital contributor social health insurance programme, the national mobile health insurance programme, as well as the voluntary participants and tertiary institutions social health insurance programme [17]. Since its introduction in 1999, Nigeria’s health insurance scheme has not been successful [16] because health care in the country is poorly funded and the health insurance system highly fragmented [18]. Thus, since its inauguration, social health insurance in Nigeria currently covers less than 5% of the country’s working population [19].

Ghana’s NHIS was promulgated in 2003 (National Health Insurance Law [Act 650 of Parliament]) but had a legal framework in 2004 (National Health Insurance Regulations [L.I.] 1809) [20,21]. The NHIS is a public health financing scheme that aims to improve access to health care for all residents of Ghana. The scheme is financed with deductions from the pension contributions of workers in the formal sector (2.5% of Social Security and National Insurance Trust [SSNIT] contributions), a 2.5% insurance levy as Valued Added Tax (VAT) on goods and services, and annual premiums paid by subscribers who are 18 years and above [22]. The scheme is also financed with voluntary contributions, donations, gifts, grants, investments, and monetary allocations made to the Health Insurance Fund (HIF) by Ghana’s legislature (parliament) [23]. Children under 18 years of age, pensioners with SSNIT, the elderly (70 years and above), pregnant women, the indigent, and Livelihood Empowerment Against Poverty (LEAP) beneficiaries, however, constitute exemptions from payment of the annual premiums [24]. From an initial coverage of 6.3% in 2005, when actual enrolment into the scheme started, the total coverage currently stands at approximately 38% [25].

Kenya has two main health insurance schemes–the National Health Insurance Fund (NHIF), established in 1966, and the National Social Security Fund (NSSF), established in 1965 [26]. Membership in the NHIF is mandatory for all workers in the formal sector but voluntary for informal sector workers. Even though the NHIF act mandates it to cover both out- and inpatient care, coverage is currently restricted to only inpatient care [27]. Aside the provision of financial security, the NSSF provides members with basic security against general illness and/or disability, employment injury, and the costs of maternity leave [26]. Under this scheme, subscribers pay premiums related to the expected cost of providing services, and it also has a community-based health insurance (CBHI), which is organised at the community level. Despite the existence of different financing schemes, health insurance currently covers 10% of Kenya’s population [28].

In Tanzania, the main provider of health insurance is the NHIF, which was established by an Act of Parliament (No. 8) in 1999 but became operational in 2001 [29]. The scheme, which was initially meant to cover only public-sector workers, currently also enrols persons in the private sector. The public-sector workers pay 3% of their monthly salaries as mandatory contributions, while the state pays an additional 3% on their behalf as their employer. Enrolment into the NHIF covers the main contributor, his or her partner/spouse, and no more than four dependents/children below 18 years of age. From an initial coverage of 2% in 2001/2002, the NHIF currently covers approximately 7.1% of the Tanzanian population (29). Across all schemes (NHIF, Social Health Insurance Benefit, Community Health Fund [CHF] and Tiba Kwa Kadi [TIKA], private insurance schemes [National insurance corporations, MEDEX (T), AAR4 health insurance, and Strategies Insurance]), however, there is a 16% level of coverage of health insurance in Tanzania [30]. Contributions by private members into the NHIF are voluntary and cover mostly salaried workers on an individual basis or as employees of registered private employers. Premiums of the private contributors are calculated based on the level of anticipated risks, such as sex, age, medical family history, and individual medical history [30].

Available evidence shows that health insurance programmes in these countries have been introduced within the last five decades and continue to evolve while striving to achieve universal health coverage [31]. Their efficiency in improving the utilisation of health care and the reduction of financial burden emanating from huge out-of-pocket expenditures for their populace is generally lacking [9,32].

The four countries were chosen for this study due their varying levels regarding health insurance coverage (Ghana: 38%, Tanzania: 16%, Kenya: 10%, and Nigeria: 3%), with the objective of understanding the variations in coverage. Ghana, Kenya, Nigeria, and Tanzania were the first sub-Saharan African countries to launch developmental plans in the early 1960s, a time that most countries in the sub-region had just gained independence from colonial rule and were preparing themselves for socio-economic expansion [33]. Inherent in these development plans was health care delivery, which, for instance, led to the introduction of health insurance in Kenya and a free health care policy for the inhabitants in Ghana [34,35].

Even though some studies have been conducted at the individual country level [16,23,25,26,27–30,36], the only study found to have been conducted in all four countries was by Carapinhaa, Ross-Degnan, Destac, and Wagner [37], which focused on the medical benefits of health insurance. There is, thus, a paucity of empirical literature on the variations that exist in health insurance coverage in the four countries. Our study, therefore, examined the variations in health insurance coverage in Ghana, Kenya, Nigeria, and Tanzania, with the objective of making policy suggestions that seek to improve upon the implementation of the various schemes by their managers.

## Materials and methods

We used data from demographic and health surveys (DHS) of Ghana (2014), Kenya (2014), Nigeria (2013), and Tanzania (2015) for this paper. DHS are nationwide surveys designed and conducted every five years in developing countries across the globe. The surveys mainly focus on maternal and child health and are designed to provide adequate data for monitoring the demographics and health conditions in developing countries. The data are specifically collected on maternal and child health outcomes, non-communicable diseases, fertility, physical activity, alcohol consumption, sexually transmitted infections, health insurance, and tobacco use. The surveys from which we drew data for this study were carried out by the Ghana Statistical Service (GSS), the Kenyan National Bureau of Statistics (KNBS), the National Population Commission of the Federal Republic of Nigeria, and the National Bureau of Statistics, Dar es Salaam in Ghana, Kenya, Nigeria, and Tanzania, respectively. All the surveys were conducted with technical support from ICF International through the MEASURE DHS programme. The demographic and health surveys were conducted among women of reproductive age (15–49 years) and productive men (15–59). Ethical approval for DHS is usually acquired from the ethics regulatory bodies of the various countries for the studies to be conducted.

In the 2014 Ghana DHS, 9396 women aged 15–49 and 4388 men aged 15–59 from 12,831 households were interviewed throughout Ghana. In Kenya, 31,079 women and 12,818 men from 40,300 households were interviewed, while 39,948 women and 17,359 men from 38,522 households were interviewed in Nigeria. In Tanzania, 13,266 women and 3,512 men were interviewed. For the purpose of this study, the samples used were 9,378 women and 4,371 men for Ghana, and 14,656 women and 12,712 men for Kenya. For Nigeria, 38,598 women and 17,185 men were included, while 10,123 women and 2,514 men were used for the Tanzanian analysis. The men and women used in our analysis are those who provided responses to the question asked in relation to the outcome variable: ‘covered by health insurance’. Permission to use the data set was given by the MEASURE DHS following the assessment of a concept note. The data are available to the public at: Ghana: https://dhsprogram.com/data/dataset/Ghana_Standard-DHS_2014.cfm?flag=0; Kenya: https://dhsprogram.com/data/dataset/Kenya_Standard-DHS_2014.cfm?flag=1; Nigeria: https://dhsprogram.com/data/dataset/Nigeria_Standard-DHS_2013.cfm?flag=1; and Tanzania: https://dhsprogram.com/data/dataset/Tanzania_Standard-DHS_2015.cfm?flag=1

The outcome variable employed in this paper was ‘covered by health insurance’. It was coded as 1 = “Yes” and 0 = “No”. Age, level of education, residence, wealth status, and occupation were the explanatory variables. Our choice of the five explanatory variables was influenced by variables included in the DHS datasets and previous studies that found these variables to be important socio-economic variables influencing health care service utilisation [38–42]. Age for females was categorised into 15–19, 20–24, 25–29, 30–34, 35–39, 40–44, and 45–49 years (women of reproductive age). The age of males was categorised as 15–19, 20–24, 25–29, 30–34, 35–39, 40–44, 45–49, 50–54, and 55–59 years (sexually active and productive men). Data were not available for males aged 50–54 or 55–59 years in Tanzania and Nigeria, respectively, nor were they available for males aged 55–59 years in Kenya. In our analysis, we separated the males from females because the DHS files were separated by sex, and, in the literature, ownership of insurance varies by sex. Educational level was separated into four categories: no education, primary level, secondary level, and higher education. Residence was categorised as rural and urban, while wealth status was grouped into poorest, poorer, middle, richer, and richest. Occupation was also placed into eight groups: not working, professional, clerical, sales, agriculture, services, skilled, and unskilled. There were no data on sales for Kenya or Tanzania.

Descriptive and inferential statistics were used to analyse the data. The descriptive statistics comprised frequencies and percentages presented in the form of tables and line graphs, while the inferential statistics adopted were bivariate and multivariate analysis. The bivariate analysis was performed using chi-square, and the multivariate analysis was performed using binary logistic regression. The logistic regression model was used to investigate the relationship between the explanatory variables and the outcome variable. The acceptable level of significance for the inferential statistics was p<0.05. To make the findings representative, both the descriptive and inferential analyses were weighted using the probability weighted variable (v005). STATA version 13 (by StataCorp located at College Station, USA) was used to run all the analyses. All analysis was done using the women files and male files separately since they were both captured in different files.

## Results

**Fig 1:**
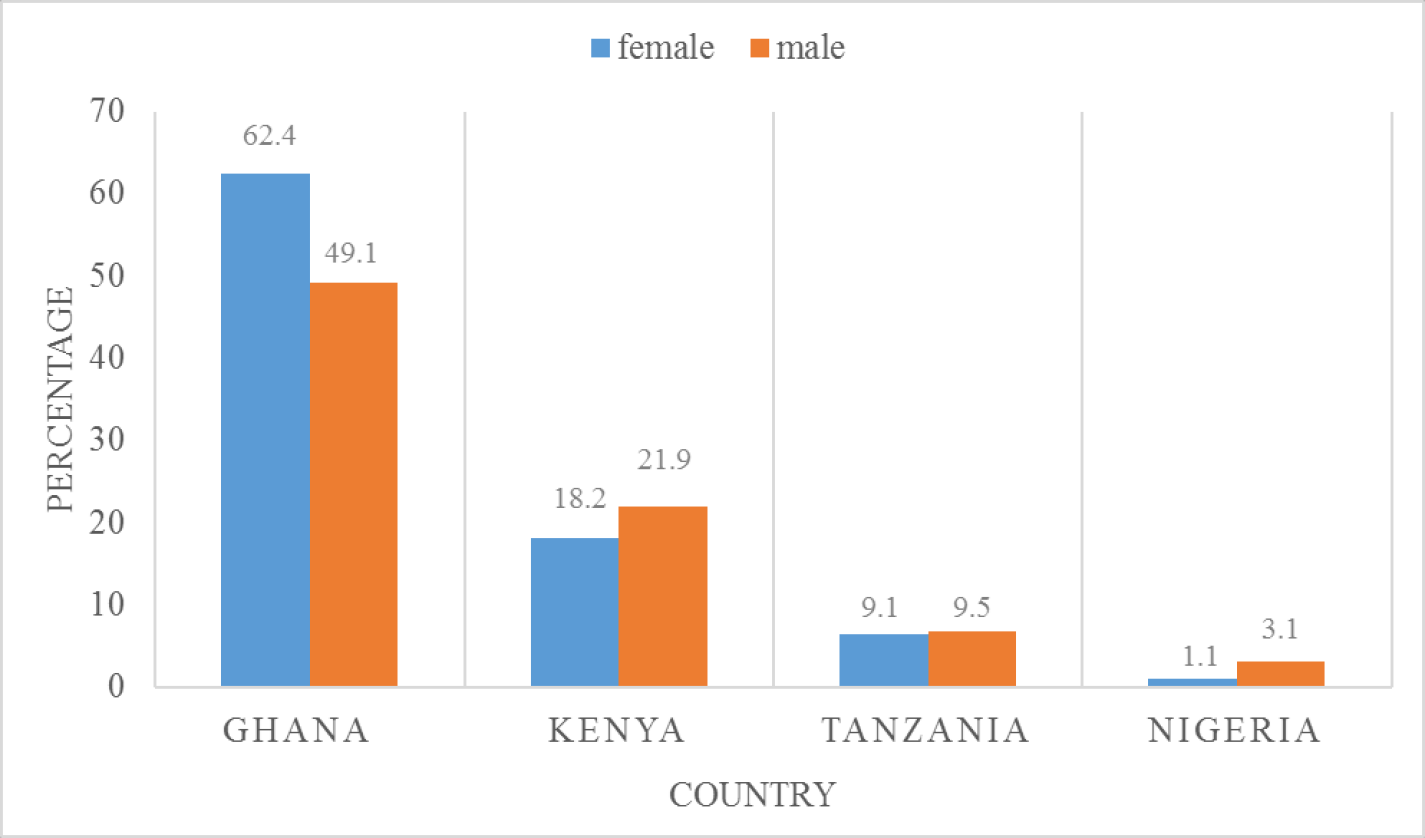
Health insurance coverage in Ghana, Kenya, Tanzania and Nigeria.

Fig 1 presents the health insurance coverage in the four countries, Ghana, Kenya, Nigeria, and Tanzania. The highest coverage was recorded in Ghana (Females = 62.4% and Males = 49.1%) and was followed by Kenya, with 21.9% and 18.2% among females and males, respectively. Tanzania also recorded a coverage of 9.5% among males and 9.1% among females. Nigeria was the least in terms of coverage, with 3.1% for males and 1.1% for females. We also observed that, apart from Ghana, coverage was higher among males than females (Fig 1).

Table 1 presents the proportions of health insurance coverage among females in Ghana, Kenya, Tanzania, and Nigeria, while Table 2 presents the coverage among males. Coverage was highest among Ghanaian males in their late thirties (56.4%). For females in Ghana, however, those aged 30–34 and 35–39 years together recorded the highest proportion of coverage, at 66.2%. In Tanzania, the highest proportion of coverage was among males in their late 40s (14.9%). On the other hand, coverage among females in Tanzania increased with age (see Table 1). Coverage among males in Nigeria increased with age, while females in their 30s and early 40s recorded the highest proportion of coverage (2.6%).

**Table 1:** Socio-economic characteristics of respondents and health insurance coverage (females).

Variables	Ghana		Kenya		Nigeria		Tanzania	
	N (%)	X^2^ (p value)	N (%)	X^2^ (p value)	N (%)	X^2^ (p value)	N (%)	X^2^ (p value)
**Age**		42.6 (p<0.001)		337 (p<0.001)		100.3 (p<0.001)		73.5 (p<0.001)
15–19	956 (59.4)		207 (7.6)		70 (0.9)		204(7.0)	
20–24	934 (58.0)		359 (13.3)		118 (1.8)		173(7.0)	
25–29	1055 (65.9)		669 (22.8)		128 (1.8)		161(7.6)	
30–34	905 (66.2)		491 (22.7)		147 (2.6)		171(9.8)	
35–39	854 (66.2)		420 (23.5)		123 (2.6)		165(10.0)	
40–44	614 (59.7)		299 (23.1)		95 (2.6)		183(13.5)	
45–49	521 (60.8)		219 (20.5)		58 (1.7)		142(14.3)	
**Level of education**		66.4 (p<0.001)		2.1 (p<0.001)		1.9 (p<0.001)		418.9 (p<0.001)
No education	1102 (61.6)		25(2.4)		30 (0.2)		90(4.6)	
Primary	940 (56.3)		733(10.0)		42 (0.6)		571(7.0)	
Secondary	3362 (63.1)		1008(21.4)		277 (2.0)		451(15.4)	
Higher	445 (74.9)		894 (56.4)		384(10.9)		89(48.4)	
**Residence**		15.8 (p<0.001)		272 (p<0.001)		383.7 (p<0.001)		39.9 (p<0.001)
Urban	3202 (63.6)		1518(25 .6)		561 (3.5)		497(10.3)	
Rural	2647 (61.0)		1142(13.1)		172 (0.8)		703(8.3)	
**Wealth status**		46.5 (p<0.001)		1.7 (p<0.001)		1.3 (p<0.001)		208.3 (p<0.001)
Poorest	975 (64.5)		63 (2.8)		2 (0.02)		92(4.1)	
Poorer	941 (57.7)		163 (6.3)		9 (0.1)		122(5.4)	
Middle	1139 (58.9)		367 (12.8)		50 (0.7)		188(8.1)	
Richer	1302 (61.7)		669 (21.5)		139 (1.8)		277(9.8)	
Richest	1491 (68.1)		1399(36.4)		533 (6.0)		521(14.5)	
**Occupation**		49 (p<0.001)		1.3 (p<0.001)		1.5 (p<0.001)		649.7 (p<0.001)
Not working	1390 (63.2)		493 (10.0)		197 (1.4)		268(8.8)	
Professional	391 (74.4)		692 (49.7)		221 (12.1)		201(47.1)	
Clerical	82 (71.2)		66.4(59 .7)		26 (10.4)		18(23.5)	
Sales	2102 (61.1)	-	-	-	139 (1.1)		-	
Agriculture	1054 (60.2)		385 (12.6)		12 (0.3)		412(7.2)	
Services	133 (66.9)		289 (20.8)				56(10.2)	
Skilled	602 (60.4)		505 (18.4)		38 (1.0)		82(6.8)	
Unskilled	96 (63.2)		229 (23.0)		100 (5.4)		163(7.9)	

Computed from 2014 GDHS, 2014 KDHS, 2015 TDHS, and 2013 NDHS

N = Samples covered with health insurance

**Table 2:** Socio-economic characteristics of respondents and health insurance coverage (males).

Variables	Ghana		Kenya		Nigeria		Tanzania	
	N (%)	X^2^ (P-value)	N (%)	X^2^ (P-value)	N (%)	X^2^ (P-value)	N (%)	X^2^ (P-value)
**Age**		51.4 (p<0.001)		146 (p<0.001)		524.6 (p<0.001)		19.5 (p<0.01)
15–19	464 (54.4)		39 (1.1)		224 (9.0)		66(7.1)	
20–24	248 (42.3)		57 (2.0)		227 (13.2)		49(8.4)	
25–29	233 (39.9)		67 (2.4)		507 (24.3)		45(9.3)	
30–34	237 (43.0)		100 (4.2)		537 (30.2)		44(10.7)	
35–39	263 (56.4)		95 (4.4)		405 (27.4)		43(9.3)	
40–44	224 (49.2)		96 (5.4)		376 (30.8)		42(12.6)	
45–49	197 (55.9)		88 (5.1)		233 (29.4)		47(14.9)	
50–54	158 (52.3)		-		221 (29.2)		-	
55–59	120 (55.0)		-		-		-	
**Level of education**		131 (p<0.001)		1.0 (p<0.001)		1.0 (p<0.001)		268.1 (p<0.001)
No education	215 (45.8)		3 (0.1)		13 (3.2)		17(6.0)	
Primary	210 (35.7)		21 (0.7)		721 (11.8)		119(5.3)	
Secondary	1347 (48.2)		188 (2.3)		1077(24.3)		144(16.1)	
Higher	372 (72.1)		325 (13.3)		969 (55.5)		55(56.3)	
**Residence**		28.2 (p<0.001)		209 (p<0.001)		271.0 (p<0.001)		11.0 (p<0.001)
Urban	1173(51.6)		388 (5.1)		1697(30.7)		133(10.7)	
Rural	972 (46.4)		154 (1.6)		1083(15.1)		201(8.9)	
**Wealth status**		51.3 (p<0.001)		757.9 (p<0.001)		1.5 (p<0.001)		90.1 (p<0.001)
Poorest	359 (47.8)		0 (0)		65 (3.6)		23(3.9)	
Poorer	337 (43.4)		12 (0.4)		220 (9.8)		38(6.6)	
Middle	346 (41.6)		47 (1.4)		384 (15.3)		31(4.7)	
Richer	481 (50.5)		97 (2.5)		793 (25.4)		95(12.5)	
Richest	619 (58.7)		385 (8.9)		1317 (43.4)		148(16.1)	
**Occupation**		186.2 (p<0.001)		1.2 (p<0.001)		1.6 (p<0.001)		287 (p<0.001)
Not working	372 (62.1)		73 (2.1)		178 (8.6)		50(12.5)	
Professional	369 (70.0)		227 (14.5)		818 (53.7)		73(48.5)	
Clerical	46 (58.7)		24 (16.9)		76 (72.8)		13(57.0)	
Sales	159 (40.6)		23 (0.9)		-		-	
Agriculture	609 (43.9)		7 (0.2)		330 (12.2)		128(7.0)	
Services	63.6(62.5)		111 (11.8)		246 (34.4)		19(14.4)	
Skilled	309 (41.7)		61 (1.9)		742 (23.0)		25(3.9)	
Unskilled	217 (40.0)		11 (1.7)		389 (16.5)		27(7.9)	

Computed from 2014 GDHS, 2014 KDHS, 2015 TDHS, and 2013 NDHS

N = Samples covered with health insurance

The proportion of health insurance coverage in all four countries for both males and females was higher among those living in urban areas than the rural dwellers. Both males and females from the richest households in all four countries also recorded the highest proportions of coverage. Males and females from the poorest households in all the countries, apart from Ghana, were the least covered. Males and females in the professional working class in Ghana and clerical workers in Kenya and Tanzania had the highest coverage rates. For those in Nigeria, while the highest proportion of health insurance coverage for males was recorded among clerical workers (16.9%), those in the professional class recorded the highest proportion of coverage among females (10.4%). The chi-square tests conducted showed that all the explanatory variables, i.e., age, level of education, residence, wealth status, and occupation, significantly related to health insurance coverage among males and females in all four countries.

Tables 3 and 4 present the results of the binary logistic regression analysis for the variations of insurance coverage in the four countries among females and males. In Ghana, women aged 30–34 and 35–39 years had the highest probability of being covered by health insurance (OR = 1.53, 95% CI = 1.29–1.81). Among the males in Ghana, the highest probability of being covered by health insurance was experienced among those in their late 30s (OR = 1.19, 95% CI = 0.92–1.54), while those in their early 30s had a lower probability of being covered by health insurance compared to those in their late teens (OR = 0.67, 95% CI = 0.46–0.75). We found that among males in Kenya and females in Tanzania, the probability of health insurance coverage increased with age. However, no discernible patterns in coverage for age were observed in Nigeria, among males in Tanzania, or among Females in Kenya.

**Table 3:** Multivariate logistic regression on health insurance coverage (females).

Variables	Ghana OR (CI)	Kenya OR (CI)	Nigeria OR (CI)	Tanzania OR (CI)
**Age**				
15–19	Ref	Ref	Ref	Ref
20–24	1.01(0.87–1.17)	1.06(0.85–1.32)	1.62**(1.19–2.20)	0.97(0.77–1.22)
25–29	1.48***(1.26–1.74)	2.16***(1.75–2.66)	1.55**(1.13–2.13)	1.06(0.83–1.36)
30–34	1.53***(1.29–1.81)	2.55***(2.05–3.17)	2.46***(1.78–3.89)	1.57***(1.23–2.02)
35–39	1.53***(1.29–1.81)	2.97***(2.38–3.71)	2.69***(1.92–3.76)	1.73***(1.34–2.22)
40–44	1.32**(1.11–1.59)	3.13***(2.47–3.97)	2.89***(2.02–4.12)	2.40***(1.87–3.09)
45–49	1.42***(1.17–1.72)	2.84***(2.21–3.65)	2.40***(1.64–3.51)	2.46***(1.91–3.09)
***Level of education***				
No education	Ref	Ref	Ref	Ref
Primary	0.94(0.82–1.08)	2.78***(1.96–3.95)	1.14(0.73–1.77)	2.00***(1.54–2.60)
Secondary	1.22**(1.07–1.38)	5.56***(3.89–7.92)	2.00***(1.36–2.95)	3.05***(2.27–2.21)
Higher	1.77***(1.32–2.36)	14.69***(10.11–21.35)	4.81***(3.21–7.21)	7.93***(4.89–12.87)
***Residence***				
Urban	Ref	Ref	Ref	Ref
Rural	0.88**(0.78–0.99)	1.28***(1.13–1.45)	1.02(0.86–1.22)	1.15(0.97–1.36)
***Wealth status***				
Poorest	Ref	Ref	Ref	Ref
Poorer	0.73***(0.64–0.84)	2.01***(1.50–2.69)	3.00*(0.83–10.83)	1.35**(1.00–1.81)
Middle	0.68***(0.59–0.79)	3.74***(2.84–4.92)	12.25***(3.75–40.05)	1.67***(1.27–2.21)
Richer	0.68***(0.58–0.81)	6.43***(4.91–8.44)	20.57***(6.30–67.13)	2.18***(1.65–2.87)
Richest	0.78**(0.64–0.95)	10.38***(7.82–13.77)	46***(14.08–151.32)	2.90***(4.89–12.87)
***Occupation***				
Not working	Ref	Ref	Ref	Ref
Professional	1.22(0.93–1.61)	2.45***(1.86–2.71)	1.87***(1.49–2.34)	3.80***(2.89–5.00)
Clerical	0.91(0.56–1.46)	2.79***(1.75–4.44)	1.49*(0.93–2.39)	1.75*(0.98–3.11)
Sales	0.81**(0.71–0.92)	-	0.60***(0.47–0.76)	-
Agriculture	0.73***(0.63–0.85)	1.37**(1.15–1.63)	0.47**(0.28–0.78)	1.18(0.96–1.45)
Services	1.05(0.72–1.52)	1.56***(1.29–1.90)	-	1.17(0.84–1.64)
Skilled	0.85*(0.72–1.00)	1.38**(1.13–1.59)	0.83(0.59–1.16)	0.63**(0.47–0.84)
Unskilled	0.82(0.56–1.19)	2.05***(1.64–2.56)	1.69***(1.29–2.20)	0.74**(0.59–0.93)

*p<0.10

**p<0.05

***p<0.001

OR = Odds Ratio CI = Confidence Interval Ref = Reference category

Computed from 2014 GDHS, 2014 KDHS, 2015 TDHS, and 2013 NDHS

**Table 4:** Multivariate logistic regression on health insurance coverage (males).

Variables	Ghana OR (CI)	Kenya OR (CI)	Nigeria OR (CI)	Tanzania OR (CI)
**Age**				
15–19	Ref	Ref	Ref	Ref
20–24	0.73**(0.58–0.91)	0.63***(0.50–0.80)	1.18(0.77–1.80)	1.16(0.75–1.79)
25–29	0.59***(0.46–0.75)	1.23*(0.98–1.56)	1.11(0.71–1.72)	1.07 (0.64–1.78)
30–34	0.67**(0.52–0.87)	2.01***(1.59–2.54)	1.86**(1.20–2.90)	1.54*(0.93–2.57)
35–39	1.19 (0.92–1.54)	2.07***(1.63–2.63)	1.98**(1.26–3.13)	1.61*(0.97–2.67)
40–44	0.93 (0.72–1.21)	2.51***(1.97–3.20)	2.73***(1.72–4.33)	2.18**(1.30–3.65)
45–49	1.16 (0.88–1.54)	2.61***(2.01–3.38)	2.90***(1.83–4.62)	2.68***(1.60–4.48)
50–54	1.08 (0.81–1.44)	2.89***(2.21–3.76)	-	-
55–59	1.07 (0.78–1.48)	-	-	-
***Level of education***				
No education	Ref	Ref	Ref	Ref
Primary	0.77**(0.61–0.97)	3.30***(1.97–5.55)	4.16*(0.96–18.09)	1.10(0.59–2.05)
Secondary	1.15(0.94–1.41)	6.82***(4.05–11.49)	8.30**(2.01–34.34)	2.18**(1.12–4.27)
Higher	2.62***(1.86–3.68)	17.19***(10.09–29.28)	5.17***(5.17–88.80)	6.02***(2.54–14.25)
***Residence***				
Urban	Ref	Ref	Ref	Ref
Rural	0.86**(0.71–0.99)	1.18**(1.04–1.34)	1.05(0.84–1.30)	1.51**(1.08–2.13)
***Wealth status***				
Poorest	Ref	Ref	Ref	Ref
Poorer	0.71***(0.59–0.86)	2.47***(1.89–3.23)	2.70(0.33–22.27)	2.21**(1.22–4.00)
Middle	0.66***(0.53–0.82)	3.67***(2.83–475)	8.14**(1.10–60.30)	1.47(0.79–2.72)
Richer	0.73**(0.57–0.93)	5.71***(4.43–7.37)	11.49**(1.56–84.62)	3.69***(2.10–6.50)
Richest	0.74**(0.56–0.99)	8.74***(6.69–11.42)	27.30**(3.71–201.14)	4.22***(2.24–7.95)
***Occupation***				
Not working	Ref	Ref	Ref	Ref
Professional	0.87(0.63–1.20)	3.80***(2.96–4.87)	1.94***(1.38–2.73)	2.19**(1.19–4.04)
Clerical	0.67(0.37–1.20)	8.33***(4.84–14.34)	2.72**(1.54–4.81)	1.75(0.63–4.85)
Sales	0.49***(0.36–0.66)	-	0.32***(0.19–0.53)	-
Agriculture	0.46***(0.36–0.58)	1.39**(1.09–1.78)	0.20***(0.10–0.41)	0.75(0.47–1.19)
Services	0.74(0.46–1.18)	3.08***(2.33–4.09)	3.94***(2.77–5.59)	1.19(0.64–4.85)
Skilled	0.44***(0.34–0.57)	1.99***(1.56–2.52)	0.68*(0.46–1.00)	0.30***(0.17–0.54)
Unskilled	0.43***(0.35–0.57)	1.33**(1.03–1.71)	0.71(0.33–1.53)	0.46**(0.25–0.85)

*p<0.10

**p<0.05

***p<0.001

OR = Odds Ratio CI = Confidence Interval Ref = Reference category

Computed from 2014 GDHS, 2014 KDHS, 2015 TDHS, and 2013 NDHS

Apart from females in Nigeria, the probability of being covered by health insurance was highest for both males and females with higher education in all four countries (See Tables 3 & 4). For instance, males with higher education in Kenya were 17.19 times more likely to be covered by health insurance than those with no education. The probability of health insurance coverage also increased with the level of education among males in Kenya, Tanzania, and Nigeria and among females in Kenya and Tanzania (See Tables 3 & 4). Whereas the probability of being covered by health insurance was lower among rural dwellers in Ghana than among those living in urban areas, the reverse was observed in the rest of the countries, where rural dwellers were more likely to be covered than those living in urban areas.

The probability of being covered by health insurance increased with wealth in Kenya and Nigeria and among males in Tanzania. For instance, in Nigeria, females and males in the richest wealth quintiles were 46 and 27.30 times, respectively, more likely to be covered than those in the lowest wealth quintiles. In Ghana, however, males and females in the poorest households were more likely to be covered compared to those in higher wealth quintiles. Apart from females in Ghana, where all of those in active employment were less likely to be covered compared to the unemployed, no discernible pattern was found in the probability of health insurance coverage by occupation.

## Discussion

Coverage of health insurance in Ghana was the highest, while that of Nigeria was the lowest. This stems from the fact that the level of importance attached to the financing of health insurance in Ghana is higher than that in Nigeria [5] as well as the other countries included in our analysis. For instance, with an estimated population of 28,206,728, [43] Ghana’s public expenditure on health in 2016 was 59.8% [44] of the country’s total public expenditure. With estimated populations of 185,989,640, 48,461,567, and 55,572,201, Nigeria, Kenya, and Tanzania, respectively, have 25.1%, 12.8%, and 46.41% of their public expenditure going to the health sector [43,44]. This partly explains why coverage was highest in Ghana.

Ghana’s highest coverage among the four countries may be attributed to the fact that it has one harmonised public health insurance scheme, which ensures risk pooling and increases the confidence of potential subscribers in the system, hence encouraging them to enrol. Furthermore, the scheme is highly pro-poor, which makes it possible for all indigents to enrol without paying the required annual premiums [25]. Financial contributions to the NHIS in Ghana are designed in such a way that premium payments are graded according to people's wealth status and ability to pay; the rich pay higher premiums compared to the poor [27]. This ensures that the poor are protected from the hurdle of paying high premiums. Notably, even though Ghana recorded the highest coverage in our study, it is still far from the target of universal health coverage (80%+), which was to be achieved within five years upon establishment of the scheme in 2003 [9].

Contrary to the harmonised health insurance system in Ghana, where there is one public health insurance scheme, the health insurance programmes of the other countries are highly fragmented. This likely negatively affected resource pooling and inefficiency on the part of the schemes and created a sense of uncertainty about benefits among potential subscribers. Combined, this may have prevented them from enrolling into the schemes, especially in Tanzania and Nigeria, which recorded the two lowest coverage rates [45]. The low coverage in Kenya, Tanzania, and Nigeria could also be attributed to cumbrous claiming processes and poor quality services provided in accredited health facilities [28,46].

Our finding that those in the highest wealth quintiles in Kenya, Tanzania, and Nigeria were the most likely to be covered by health insurance points to the effects of wealth variations in health insurance coverage and confirms arguments that, even though health insurance schemes in sub-Saharan African countries are designed to be pro-poor in nature and help ease the financial burden on the poorest households, the majority of subscribers are those in the upper wealth quintiles as the poor are unable to enrol [47,48]. The findings are in contradiction with postulations regarding risk aversion and health insurance coverage among the poor. The World Bank [49] and Wagstaff [50], for instance, suggest that households tend to be progressively more averse to risk as they get nearer to poverty, and this is because continuous dips in wealth status have the propensity to push them below survival points. Poor households, which are more likely to have financial challenges in the future, are therefore more likely to sacrifice their present incomes and subscribe to health insurance to reduce future risk [49–51]. The fact that the poorest in Ghana were, however, the most likely to be covered may be credited to the exemptions policy of the country’s health insurance scheme, where the indigent and LEAP beneficiaries are exempted from paying the annual premiums required for coverage, as posited by Duku et al. [24].

Our findings also point to the important role of female education in positively influencing the health decisions of women. For example, aside from females in Nigeria, the likelihood of being covered by health insurance was highest for persons with the highest levels of education in all the countries. This, therefore, is a justification of the Commission on Social Determinants of Health’s [52] argument that education enables women to protect their own health and to seek appropriate health care when they are ill. Thus, being covered by health insurance enables them to avoid catastrophic health expenditures that they would have to make out-of-pocket when they fall ill and do not have health insurance [53,54], and being educated makes it possible for them to have that foresight and insure themselves against the unexpected out-of-pocket payments [55,56].

### Limitations

Despite the relevance of the findings in this study, it is important to indicate the potential limitations inherent in the study. Demographic and health surveys use a cross-sectional design, which made it impossible for us to account for unobserved heterogeneity. There are also limitations that come with self-reporting by participants of the surveys, such as recall bias or deliberate misreporting. Nonetheless, the strengths of our findings are rooted in the study design, data collected using standard methodologies, and sample yield that was comparable across countries.

## Conclusions

Kenya, Tanzania, and Nigeria might not be able to achieve universal health coverage and meet some of the sustainable development goals on health by the year 2030 if the current health insurance financing mechanisms persist. For the insurance schemes in Kenya, Tanzania, and Nigeria to increase their coverage and achieve universal health coverage, the various schemes should be harmonised into single health financing schemes. This would help to maximise the size of their risk pools and increase the confidence of potential subscribers in the system. Fundamentally, female education should be given more attention since education among females was found to be a strong factor influencing health insurance coverage.
